# Genome Sequencing Reveals the Adaptation of Chickens to High Altitudes in Different Regions

**DOI:** 10.3390/ani15020265

**Published:** 2025-01-18

**Authors:** Yizhou Hu, Xing Li, Qixin Guo, Lan Huang, Hao Bai, Guobin Chang

**Affiliations:** 1 Key Laboratory of Animal Genetics and Breeding and Molecular Design of Jiangsu Province, College of Animal Science and Technology, Yangzhou University, Yangzhou 225009, China; huyizhou615@163.com (Y.H.); mx120210885@stu.yzu.edu.cn (X.L.); dx120190114@yzu.edu.cn (Q.G.); dx120200133@stu.yzu.edu.cn (L.H.); 2 Joint International Research Laboratory of Agriculture and Agri-Product Safety, The Ministry of Education of China, Institutes of Agricultural Science and Technology Development, Yangzhou University, Yangzhou 225009, China; bhowen1027@yzu.edu.cn

**Keywords:** genome sequencing, altitudinal adaptation, chicken

## Abstract

Altitudinal adaptation is a key factor in species formation and leads to increased species diversity. Chickens are one of the most widely distributed and important domesticated species, making them ideal models for studying the evolution of altitudinal adaptation. Therefore, we downloaded and analyzed the total genome data of 160 individual chickens from seven sampling regions at two different altitudes (>3000 m and <600 m). In total, 21,672,487 high-quality single-nucleotide polymorphisms were selected and used for subsequent analyses. First, we interpreted the genetic relationships among chickens from different sampling regions using a neighbor-joining tree, population structure, and four dimensionality reduction methods. We found that 38 genes were significantly associated with altitudinal adaptations by *F*_ST_ and θπ. Functional annotation of the genes showed that they are primarily involved in energy metabolism, ion channel activity, and blood pressure regulation. Our results provide evidence of genetic diversity among different chicken breeds and reveal the mechanisms of adaptation to high altitudes.

## Simple Summary

High-altitude adaptation is a key factor in species formation, leading to increased species diversity. Chickens are one of the most widely distributed and important domesticated species, making them ideal models for studying the evolution of altitudinal adaptation. We downloaded and analyzed the total genome data of 160 individual chickens from seven sampling regions at two different altitudes (>3000 m and <600 m), from which we selected 21,672,487 high-quality single-nucleotide polymorphisms (substitutions of a single nucleotide at a specific position in the genome) for analysis. First, we interpreted the genetic relationships among chickens from different sampling regions using a neighbor-joining tree, population structure, and four dimensionality reduction methods, and we found that 38 genes were significantly associated with altitudinal adaptations. Functional annotation of the genes showed that they are primarily involved in energy metabolism, ion channel activity, and blood pressure regulation. Our results provide evidence of genetic diversity among different chicken breeds and reveal the mechanisms of adaptation to high altitudes.

## 1. Introduction

Chickens (*Gallus domesticus*) are an important food source in many cultures worldwide. Compared to other domestic animals such as sheep, cattle, and pigs, chickens are currently the preferred and most abundant source of animal protein globally [1]. Across cultures, religions, and societies, chickens are widely accepted, with few or no taboos compared to other domestic animals. They also have a high potential for adaptation to different environmental conditions and genetic improvements during breeding. They are important model animals for biomedical research [2,3]. Chickens are widely distributed across nearly every continent and are highly adaptable to various environments.

Adaptation to high-altitude environments has recently emerged as an interesting research topic. Furthermore, studying the adaptation to harsh environments and the pathogenesis of hypoxia-related diseases is crucial. Adaptation to harsh environments is a complex process involving biological pathways and gene networks [4]. Studies have identified genetic factors involved in high-altitude adaptation in cattle [5], sheep [6], snow leopards [7], and humans [8]. The results of these studies have shown that hypoxia is involved in cerebral edema, tumorigenesis, myocardial ischemia, and diabetes. Notably, some genes, including Integrin Subunit Alpha 7 (*ITGA7*), neurogenic locus notch homolog protein 2 (*NOTCH2*), Endothelial PAS domain-containing protein 1 (*EPAS1*), and GTP cyclohydrolase I (*GTPCH*) are critical for hypoxia adaptation [7,8,9,10]. Recent evidence indicates that positive selection in highland populations is associated with cardiovascular and respiratory system development and responses to radiation, inflammation, DNA repair, and immunity [11].

In this study, we selected 160 chickens from highland (altitude > 3000 m) and lowland (altitude < 600 m) regions. We performed a population-selective sweep analysis to identify potential genes associated with high-altitude adaptation. Our results provide valuable insights into altitude adaptation and provide a valuable genomic resource for future genome-wide analyses of economically important traits in poultry.

## 2. Materials and Methods

### 2.1. Data Resources

Whole-genome resequencing data for 160 local chickens from different altitudes were downloaded from the ChickenSD database (http://animal.omics.pro/code/index.php/ChickenVar, accessed on 7 October 2024). The dataset included low-altitude Jiangxi Silky chickens (SK, *n* = 20), Dongxiang Blue eggshell chickens (DX, *n* = 5), Anyi gray chickens (AYW, *n* = 5), Guangxi chickens (GX, *n* = 6) and India chickens (India, *n* = 49), as well as high-altitude Tibetan chickens (TB, *n* = 89) and Nixi chickens (NX, *n* = 12). The data were used to analyze the population structure, genetic diversity, and altitude adaptation of chickens at different altitudes. Table S1 lists the elevation and sampling locations of all chickens used in this study.

### 2.2. Genetic Variation Detection

First, after filtering adapters and low-quality reads from the raw resequencing data, we used the Burrows–Wheeler Aligner (BWA-MEM) to map the quality-controlled reads to the chicken reference genome (GRCg6a). Then, the alignment bam files were sorted and duplicates removed using the SortSam and MarkDuplicates tools in Picard. Next, we used the Genome Analysis Toolkit (GATK) to detect genomic variants. Specifically, we sequentially applied GATK’s HaplotypeCaller, CombineGVCFs, and GenotypeGVCFs commands to identify and merge variant sites. SNP variants were then extracted using GATK’s SelectVariants command. To obtain high-quality variant data, we applied GATK’s VariantFiltration module with the following filtering criteria: mean sequencing depth < 1/3× or mean sequencing depth > 3×; Quality by Depth (QD) < 2.0; mapping quality (MQ) < 40.0; Fisher Strand (FS) > 60.0; MQRankSum < −12.5; and ReadPosRankSum < −8.

To further ensure the validity and accuracy of subsequent analyses, we used Plink (version: 1.09) to filter for linkage disequilibrium (LD) between loci (–indep-pairwise 1000 kb 50 kb 0.2), which helps to reduce false positives and ease computational burden.

### 2.3. Phylogenetic Analysis and Principal Component Analysis

Phylogeny is the study of the evolutionary history of groups of organisms and their relationships with each other; genetic characteristics such as DNA and protein sequences can be used to judge the relationship between organisms, and the analysis results are reflected in the phylogenetic tree. After quality control of the SNPs, the genetic distance between the samples of each population was calculated using VCF2Dis software (version: 1.53, https://github.com/BGIshenzhen/VCF2Dis, accessed on 7 October 2024), and a neighbor-joining (NJ) tree was constructed according to the genetic distance using FastME [12]. R (version: 4.3.1) was used to visualize the NJ tree. Population structure analysis was performed using ADMIXTURE software (version 1.3.0, http://dalexander.github.io/admixture/, accessed on 7 October 2024) [13], assuming a gradual increase in the number of ancestral populations (K) and determining the minimum cross-validation for mapping.

In genetics, principal component analysis (PCA) is applied to genotypic data to reduce dimensionality. PCA was performed using the genome-wide complex trait analysis [14]. First, we used Plink software (version: 1.9 beta) to further filter SNPs after linkage disequilibrium filtering, with the following criteria: (1) minor allele frequency (MAF) ≥ 0.05; (2) Hardy–Weinberg equilibrium (HWE) threshold of 0.0001; and (3) SNP call rate above 95%. Next, PCA was conducted using the Python script vcf2eigenstart.py, with the significance of eigenvectors evaluated by the Tracy–Widom test. Finally, we visualized the PCA results using the ggplot2 package in R language. The neighbor-joining tree and PCA were visualized by R (version 4.3.1, https://www.r-project.org/, accessed on 7 October 2024).

### 2.4. Analysis of Population Genetic Structure

Genetic structure refers to the non-random distribution of genetic variation within a species or population. Based on geographic distribution or other criteria, a population can be divided into multiple subpopulations, with individuals within the same subpopulation being more closely related, while genetic relationships between subpopulations are more distant.

Population structure analysis of local chickens was conducted using the ADMIXTURE software (version: 1.3.0), a tool for maximum likelihood estimation of single-ancestry components from multi-locus SNP genotype datasets. ADMIXTURE employs the same statistical model as STRUCTURE but achieves faster estimates through rapid numerical optimization algorithms. Based on genomic genetic variation, ADMIXTURE calculates population stratification, determining the proportion of each individual’s genome that derives from each of the hypothesized ancestral populations. By incrementally increasing (e.g., from 1 to 10), we calculated the population structure for each value and recorded the cross-validation (CV) error for each, with the optimal value defined by the lowest CV error. Finally, the population structure results were visualized by R.

### 2.5. Dimensionality Reduction Methods Reveal the Fine Structure of Chicken Populations

The t-distributed stochastic neighbor embedding (t-SNE) method is a statistical method for visualizing high-dimensional data by assigning each data point a location on a two- or three-dimensional map. As a nonlinear dimensionality reduction technique, t-SNE maps each high-dimensional object to a point in two- or three-dimensional space, placing similar objects close together, while less similar objects are more likely to be mapped to distant points. t-SNE has been widely applied for visualization in diverse fields, including cybersecurity, cancer research, and bioinformatics.

Uniform Manifold Approximation and Projection (UMAP) is another dimensionality reduction technique, providing visualizations similar to t-SNE, but is also useful for general nonlinear dimensionality reduction. UMAP preserves the qualitative similarity of high-dimensional data in low-dimensional space by mapping the topological structure. PCA-UMAP combines PCA and UMAP, performing linear dimensionality reduction with PCA first, followed by nonlinear reduction with UMAP. Similarly, PCA-tSNE integrates PCA with t-SNE, preserving data structure and features more effectively.

To further reveal the fine structure of local chicken populations, this study applies additional nonlinear dimensionality reduction methods (t-SNE, UMAP, PCA-UMAP, and PCA-tSNE) to the SNPs and individuals after LD filtering. Dimensionality reduction based on PCA-tSNE, t-SNE, UMAP, and PCA-UMAP is conducted directly on the genotype matrix using Python modules.

### 2.6. Linkage Disequilibrium Decay

When the probability of a specific allele at one locus appearing simultaneously with a specific allele at another locus exceeds the probability expected by random distribution in the population, the two loci are said to be in a state of linkage disequilibrium (LD). LD decay is commonly represented by r^2^, which indicates the level of linkage disequilibrium within the population. In this study, we calculated the r^2^ values among multiple subpopulations based on the autosomal SNP dataset of local chicken populations using the PopLDdecay software (version: 3.43) [15]. Finally, LD decay plots for each subpopulation were generated using the Perl scripts (version: 5.40.0) provided with the software.

### 2.7. Selective Clearance Analysis

Based on the whole-genome sequences, we performed an in-depth scan of candidate genes associated with high-altitude adaptation. We divided chickens from high (TB and NX) and low (SK, DX, AYW, GX, and India) altitudes into two populations for analysis. Using only the selection signal detection method can lead to false positive selection signals; therefore, we combined two detection methods, *F_ST_* and log2 (π_high altitude_/π_low altitude_) (log2(θπ)), to avoid false positives. Notably, numerous studies have used *F_ST_* and θπ methods to detect the selective signal sweep regions in animals [16,17,18]. *F_ST_* and θπ were analyzed using VCFtools, with a 20 kb window sliding in steps of 5 kb. Putative selection targets were identified as the candidate regions in fully overlapping windows with high *F_ST_* (*F_ST_*  >  99%) and log2(θπ) (θπ  >  99%) values. *F_ST_* and π were also calculated for the different subgroups using VCF tools [19].

### 2.8. Runs of Homozygosity Analysis

Runs of homozygosity (ROHs) are formed when parents pass homozygous identical haplotypes to their offspring, and the length of the ROH fragments can be used to infer the inbreeding history. PLINK analysis of the ROHs was performed using the sliding window method with the following parameters: (1) minimum ROH length set at 500 kb, (2) interval of two consecutive SNPs < 100 kb, (3) one SNP locus was allowed to be heterozygous, (4) a minimum of 50 consecutive SNPs were included in the ROHs, (5) the proportion of homozygous overlapping windows was 0.05, and (6) minimum SNP density was set to 1 SNP per 50 kb (https://github.com/ecogenomicscanada/Runs_of_Homozygosity, accessed on 7 October 2024). Mapping was performed using R (version: 4.3.2).

### 2.9. Gene Flow Analysis

The introduction of new alleles into a population through gene exchange is a vital source of genetic variation that affects population genetic diversity. Gene flow was analyzed using Treemix software (version: 1.13 http://bitbucket.org/nygcresearch/treemix/, accessed on 7 October 2024) [20] and analyzed by R.

### 2.10. Gene Annotation and Enrichment Analysis

Candidate regions were compared with the reference genome using the National Center for Biotechnology Information (https://www.ncbi.nlm.nih.gov/, accessed on 8 October 2024) database, and genes in the candidate regions were identified as candidate genes. Based on the Gene Ontology [21] (GO) and Kyoto Encyclopedia of Genes and Genomes (KEGG) databases [22] enrichment analyses of candidate genes were performed using the KEGG Orthology Based Annotation System (KOBAS) (http://kobas.cbi.pku.edu.cn/, accessed on 8 October 2024) website [23]. The analysis files were downloaded and mapped using R (version 4.3.2).

## 3. Results

### 3.1. Analysis of Population Genetic Structure

To understand the phylogenetic relationships and genetic structure of high-altitude and low-altitude local chicken populations, we conducted phylogenetic analysis, principal component analysis, and population structure analysis based on the autosomal SNPs of 160 chickens from various altitudes.

**Figure 1:**
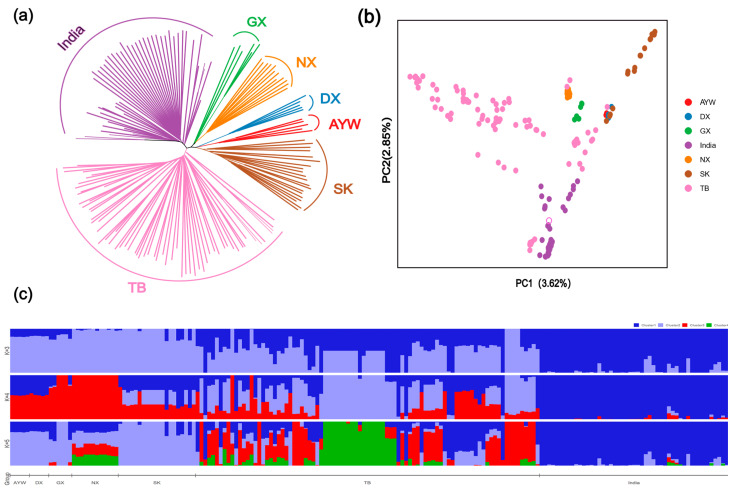
Group structure analysis. (**a**) NJ tree; (**b**) principal component analysis; (**c**) population structure analysis.

The phylogenetic tree indicates that Tibetan chickens (TB) are most closely related to Indian chickens (India) and are relatively more distantly related to Jiangxi silky chickens (SK) compared to other local chickens (Figure 1a). In population structure analysis, K is assumed to be from 2 to 10, and K = 4 is considered the most appropriate model. The results show evidence of gene flow between Tibetan chickens and other low-altitude local chickens. Additionally, Indian and Nixi chickens have relatively pure lineages, while other local chickens exhibit subpopulation admixture (Figure 1c). Overall, there is evidence of crossbreeding among most local chicken breeds. In the principal component analysis, samples from local chicken breeds, except for NX, were relatively dispersed, particularly TB, which may be due to different sampling locations and gene flow. Some TB samples overlapped with NX, GX, DX, AYW, and SK, suggesting possible crossbreeding among them (Figure 1b).

### 3.2. Different Dimensionality Reduction Methods Reveal the Fine Structure of Chicken Populations

**Figure 2:**
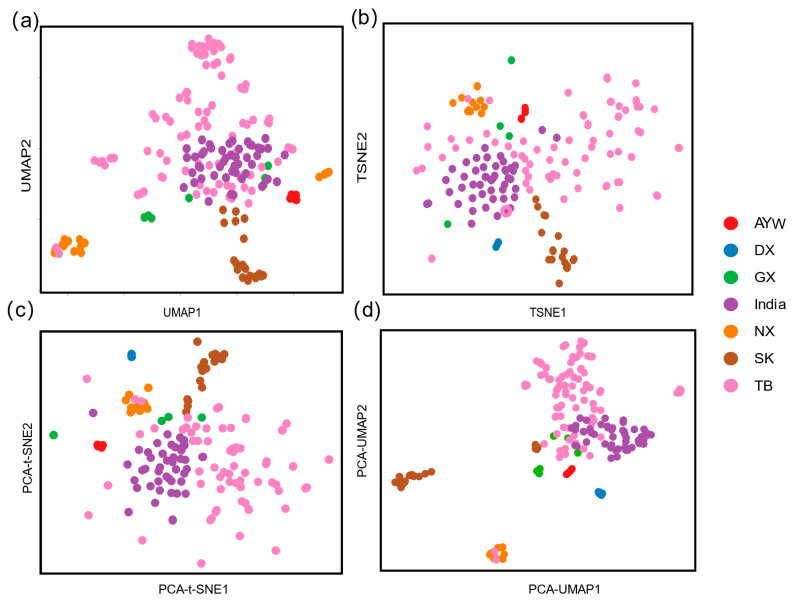
Two-dimensional visualization of population genotype data by four different downscaling methods. (**a**) UMAP dimensionality reduction method; (**b**) t-SNE dimensionality reduction method; (**c**) PCA-tSNE dimensionality reduction method; (**d**) PCA-UMAP dimensionality reduction method.

To further validate the relationship between all samples, we performed four nonlinear dimensionality reduction methods (UMAP, t-SNE, PCA-UMAP, and PCA-t-SNE) to analyze the relationships among all the chickens. The results from all four methods showed that the TB and India samples clustered together, whereas the AYW breed clustered separately from the other local chicken breeds. The PCA-UMAP results revealed that most of the SK and NX samples formed distinct clusters (Figure 2d). However, the individual distributions observed using the other dimensionality reduction methods were more difficult to distinguish. This further confirmed the accuracy of the NJ tree, population structure analysis, and PCA results. In addition, the results show that the UMAP and PCA-UMAP results are more compact, indicating that the results are more reliable than those of t-SNE and PCA-tSNE.

### 3.3. Genetic Distance and Gene Flow Analysis

**Figure 3:**
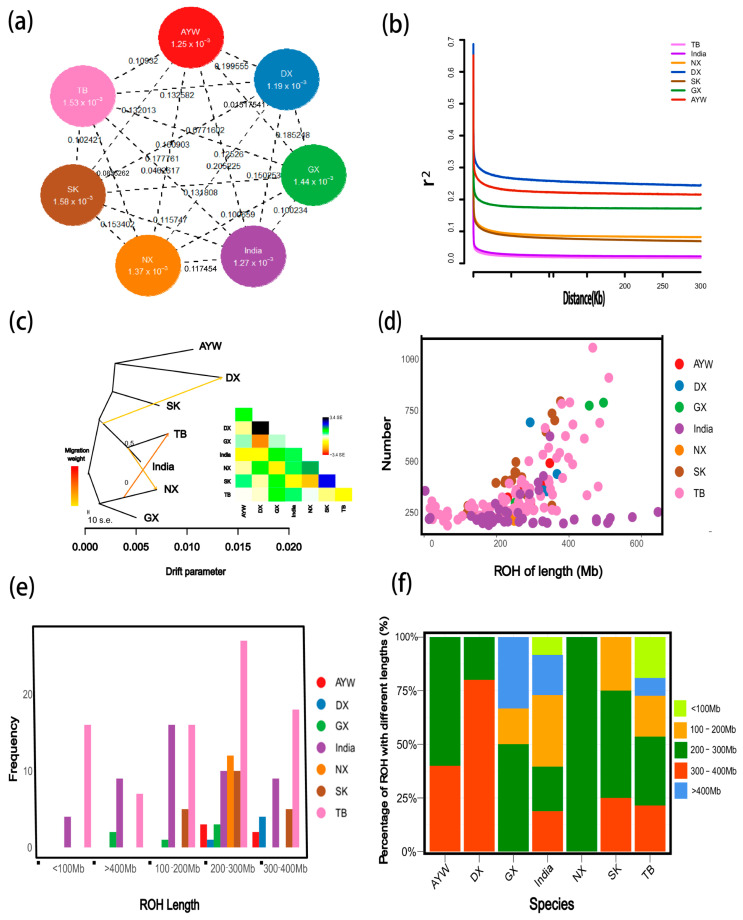
Population genetic evolution analysis of local chicken breeds. (**a**) *F_ST_* and *π* values between pairwise local chicken breeds; The values under the shadow represent *F_ST_* values, while the values connected by lines between pairs represent π values; (**b**) Linkage disequilibrium decay analysis. (**c**) Gene flow analysis; (**d**) Distribution of ROH fragment lengths and quantities in different local chicken breeds; (**e**) Number of ROH fragments in different length intervals; (**f**) Proportion of ROHs in different length ranges among various local chicken breeds.

Among the seven local chicken breeds, TB and India show the lowest level of differentiation (*F_ST_* value of 0.0462617), while TB shows the highest differentiation with DX and SK (*F_ST_* values of 0.132582 and 0.102421, respectively), further supporting the findings of the phylogenetic tree and population structure analysis. Nucleotide diversity analysis revealed that TB and SK have higher π values than the other breeds, indicating that they are less affected by selection and have higher genetic diversity (Figure 3a). To determine genetic contributions between different breeds, we performed gene flow analysis on the local chicken populations. The results indicate frequent gene flow between NX and TB, as well as between the common ancestors of TB and India with NX (Figure 3c).

### 3.4. Linkage Disequilibrium Decay

The LD decay graph shows a decrease in the average LD coefficient between molecular markers in the genome as the distance between the markers increases. The physical distance between TB populations was <50 kb when the LD coefficient decreased below 0.1, indicating a rapid decay of TB populations. Similarly, we observed that the decay rate of the LD coefficient differed among different subpopulations. The size of the decay rate among breeds followed the order (TB and India) > (SK and NX) > GX > AYW > DX, indicating that the TB population had the smallest LD decay distance (Figure 3b). This might be due to the high genetic diversity in this population, indicating that TB was less subject to selection.

### 3.5. Runs of Homozygosity

The ROH-based assessment is an approach that assesses inbreeding using information from genome-wide analyses. We performed ROH analysis on 160 chickens, and our results showed that most ROH fragments were between 150 and 300 Mb in length (Figure 3d), with >60% of ROHs having a length >200 Mb for each breed. The lowest proportion of ROHs was <100 Mb for the India breed (Figure 3f). The TB and India breeds had longer ROHs than the other varieties, suggesting a higher frequency of inbreeding in recent generations and a short population history for these two varieties (Figure 3e).

### 3.6. Genome-Wide Selection Signal Analysis

To identify candidate genes potentially associated with high-altitude adaptation in local chicken populations, we performed a genome-wide selection signal analysis based on genomic variation data. In this study, we used a combined *F_ST_* and θπ approach to screen for candidate genes likely influencing high-altitude adaptation.

**Figure 4:**
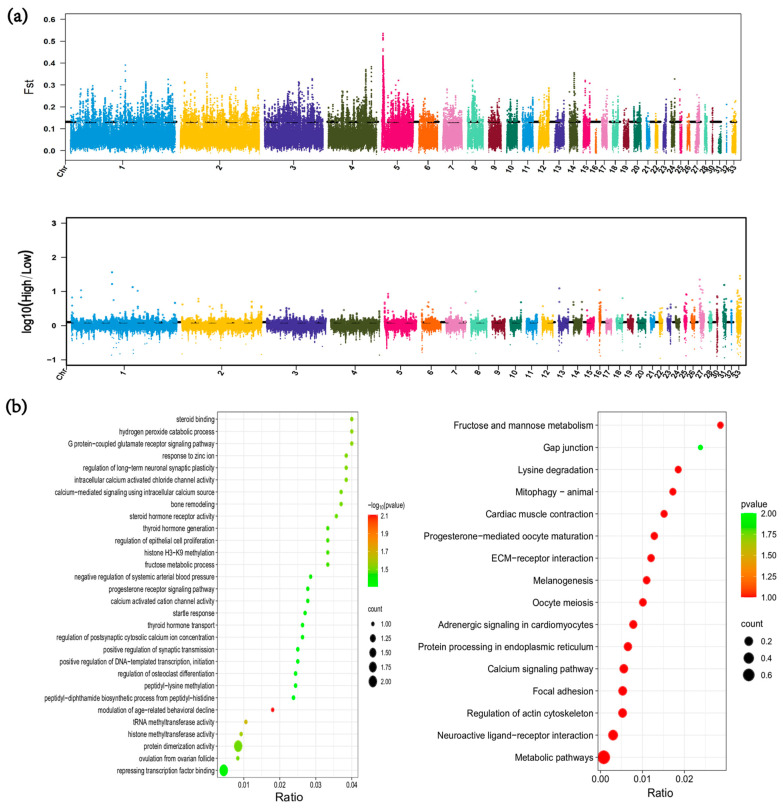
Genome-wide selective analysis of local chickens. (**a**) Selective analysis of high-altitude and low-altitude local chickens using *F_ST_* and *π-Ratio*; (**b**) Gene Ontology (GO) annotation of candidate genes (showing only 30 terms) and Kyoto Encyclopedia of Genes and Genomes (KEGG) enrichment analysis. The left panel shows GO annotation, and the right panel shows KEGG enrichment analysis.

Through *F_ST_* analysis, we selected the top 1%, identifying a total of 1694 candidate regions. Since population differentiation is influenced not only by positive selection but also by population history, we also conducted genome-wide selection signal analysis using the π-Ratio method, which is less affected by population events (Figure 4a). By selecting the top 1% in this analysis, we identified an additional 1688 candidate regions. The intersection of these two methods yielded 175 candidate regions, which were annotated to identify 38 candidate genes.

We performed GO and KEGG enrichment analyses of candidate genes in highland and lowland chicken populations. Gene Ontology enrichment revealed that these genes were mainly enriched in biological processes and molecular functions, including repression of transcription factor binding, fructose–mannose metabolism, calcium-activated channel activity, and regulation of arterial blood pressure. The KEGG analysis showed enrichment mainly in the fructose and mannose metabolism and myocardial contraction pathways. Therefore, to further identify candidate genes, we examined genes involved in energy metabolism, calcium channels, angiogenesis, and blood pressure, and identified hexokinase (*KHK*), Anoctamin-1 (*ANO1*), Myosin Heavy Chain 15 (*MYH15*), and Rho GTPase Activating Protein 42 (*ARHGAP42*).

We identified several genes associated with energy metabolism in high-altitude chickens. Notably, *KHK* is involved in pathways associated with fructose metabolism, and the fructose-driven glycolytic metabolic pathway does not require the major rate-limiting step of glucose glycolysis catalyzed by phosphofructokinase. Therefore, it enters glycolysis faster than glucose, allowing rapid energy supply to the body. Hypoxia-inducible factors (*HIFs*) are transcription factors that respond to changes in oxygen availability in the cellular environment at high altitudes. Peroxisome deficiency and HIF-2α signaling are negative regulators of *KHK* expression [24,25].

Hypoxia causes an influx of calcium (Ca^2+^) and an increase in the intracellular Ca^2+^ concentration by opening store-operated calcium (SOC) channels, resulting in increased cell contraction [26]. Simultaneously, the lack of oxygen causes vasoconstriction and increases blood pressure [27]. The *ANO1* gene activates calcium channel activity and positively regulates cellular responses to glucose stimulation, leading to insulin secretion [28,29]. *MYH15* is involved in adenosine triphosphate binding, actin filament binding, and calmodulin activity and has recently been shown to be associated with pulmonary artery blood pressure [30]. *ARHGAP42* encodes Rho GTPase-activating protein (RhoGAP) and is a member of the GTPase regulator family associated with FAK or the Bin/amphiphysin/Rvs-PH family of proteins. This gene is enriched in vascular smooth muscle cells, and its encoded protein inhibits RhoA activity to regulate vascular tone and control blood pressure [31,32].

## 4. Discussion

Although there has been research on the adaptability of different species in variable environments, a deeper understanding of the adaptation mechanisms in extreme environments is still needed [33,34,35]. In particular, high-altitude areas, with their unique ultraviolet intensity and low oxygen pressure environments, pose adaptive challenges for animals, especially chickens [36]. In order to explore the adaptation mechanism of chickens to high-altitude environments in depth, this study aims to reveal the key genes related to adaptation to high-altitude environments by analyzing the whole-genome resequencing variations of chickens in different altitude areas.

In our study, we divided seven chicken breeds into high- and low-altitude groups. Single-nucleotide polymorphism analysis showed that they could be divided into four subgroups with less differentiation between the TB and India breeds, GX breeds, and interbreeding with other breeds in low-altitude areas. This is consistent with the results of other studies [11], possibly because domesticated chickens originate from multiple regions globally [37,38].

To reveal the potential mechanisms of high-altitude adaptation, we used *F*_ST_ and nucleotide diversity methods to detect genes specifically selected between chicken breeds living at high and low altitudes. Both had strong selection signals for screening genes associated with environmental adaptation. The intersection of the two regions was used to identify 175 candidate regions, and gene annotation was performed for 38 genes. Gene Ontology and KEGG enrichment showed that they were mainly enriched in energy metabolism, ion channel activity, and blood pressure regulation. Some genes were associated with the nervous system and other cellular components. Notably, novel genes associated with high-altitude adaptation (*KHK*, *ANO1*, *MYH15*, and *ARHGAP42*) were identified. These genes are associated with high-altitude adaptation, encompassing biological functions such as energy metabolism, vasodilation, and blood pressure regulation. However, there is a lack of literature to support a direct link between these genes and high-altitude adaptation. Consequently, further validation studies are essential to ascertain the roles of these candidate genes in the context of high-altitude adaptation.

Notably, the GRM5 gene is particularly intriguing as it initiates signal transduction through guanine nucleotide-binding proteins and plays a significant role in modulating synaptic plasticity and neural network activity. Additional research has demonstrated an increase in GRM5 expression under hypoxic conditions, suggesting its potential significance in hypoxic environments [39]. Genes such as *EMILIN1*, *CGREF1*, *RIMS1*, *PRDM5*, and *PXDN* were also identified to play roles in cell adhesion, regulation of cell proliferation, neurotransmitter release, and angiogenesis, potentially influencing animal adaptation to high-altitude environments either directly or indirectly [40]. Previous studies in Tibetan sheep have shown that significant positive selection occurs for the MITF gene [41]. In our study, we similarly found positive selection for *MITF*, which is essential for pigmentation by regulating melanocyte differentiation and development. The factors driving the positive selection of *MITF* and whether it is related to high altitude adaptation are unknown, but may be related to the phenotypic evolution of Tibetan chickens.

Overall, although the roles of the high-altitude adaptation candidate genes identified here have yet to be directly confirmed in chickens, genomic-level research has laid a foundation for future molecular validation studies. Next, we will conduct more in-depth experimental research to clarify the precise roles of these genes in high-altitude adaptation.

## 5. Conclusions

In conclusion, we analyzed the whole genomes of different chicken breeds at high and low altitudes to reveal their genetic evolution between different breeds at various altitudes. We identified new genes associated with high-altitude adaptation, thereby providing a theoretical basis for elucidating the adaptation of chickens to extreme high-altitude environments.

## Data Availability

Data are contained within the article and Supplementary Materials.

## Supplementary Materials

The following supporting information can be downloaded at https://www.mdpi.com/article/10.3390/ani15020265/s1: Table S1: Sample information; Table S2: 38 identified candidate genes.
